# Predictive Computational Modeling of the Mucosal Immune Responses during *Helicobacter pylori* Infection

**DOI:** 10.1371/journal.pone.0073365

**Published:** 2013-09-05

**Authors:** Adria Carbo, Josep Bassaganya-Riera, Mireia Pedragosa, Monica Viladomiu, Madhav Marathe, Stephen Eubank, Katherine Wendelsdorf, Keith Bisset, Stefan Hoops, Xinwei Deng, Maksudul Alam, Barbara Kronsteiner, Yongguo Mei, Raquel Hontecillas

**Affiliations:** 1 Nutritional Immunology and Molecular Medicine Laboratory, Virginia Bioinformatics Institute, Virginia Tech, Blacksburg, Virginia, United States of America; 2 Center for Modeling Immunity to Enteric Pathogens Virginia Bioinformatics Institute, Virginia Tech, Blacksburg, Virginia, United States of America; 3 Network Dynamics and Simulation Science Laboratory, Virginia Bioinformatics Institute, Virginia Tech, Blacksburg, Virginia, United States of America; 4 Department of Biomedical Sciences and Pathobiology, Virginia-Maryland Regional College of Veterinary Medicine, Virginia Tech, Blacksburg, Virginia, United States of America; 5 Department of Statistics, Virginia Tech, Blacksburg, Virginia, United States of America; Immunology Frontier Research Center, Osaka University, Japan

## Abstract

T helper (Th) cells play a major role in the immune response and pathology at the gastric mucosa during *Helicobacter pylori* infection. There is a limited mechanistic understanding regarding the contributions of CD4+ T cell subsets to gastritis development during *H. pylori* colonization. We used two computational approaches: ordinary differential equation (ODE)-based and agent-based modeling (ABM) to study the mechanisms underlying cellular immune responses to *H. pylori* and how CD4+ T cell subsets influenced initiation, progression and outcome of disease. To calibrate the model, *in vivo* experimentation was performed by infecting C57BL/6 mice intragastrically with *H. pylori* and assaying immune cell subsets in the stomach and gastric lymph nodes (GLN) on days 0, 7, 14, 30 and 60 post-infection. Our computational model reproduced the dynamics of effector and regulatory pathways in the gastric lamina propria (LP) *in silico*. Simulation results show the induction of a Th17 response and a dominant Th1 response, together with a regulatory response characterized by high levels of mucosal Treg) cells. We also investigated the potential role of peroxisome proliferator-activated receptor γ (PPARγ) activation on the modulation of host responses to *H. pylori* by using loss-of-function approaches. Specifically, *in silico* results showed a predominance of Th1 and Th17 cells in the stomach of the cell-specific PPARγ knockout system when compared to the wild-type simulation. Spatio-temporal, object-oriented ABM approaches suggested similar dynamics in induction of host responses showing analogous T cell distributions to ODE modeling and facilitated tracking lesion formation. In addition, sensitivity analysis predicted a crucial contribution of Th1 and Th17 effector responses as mediators of histopathological changes in the gastric mucosa during chronic stages of infection, which were experimentally validated in mice. These integrated immunoinformatics approaches characterized the induction of mucosal effector and regulatory pathways controlled by PPARγ during *H. pylori* infection affecting disease outcomes.

## Introduction

*Helicobacter pylori* is a Gram-negative, microaerophilic bacterium of the Epsilonproteobacteria that colonizes the stomach of nearly a half of the world’s population. The presence of *H. pylori* in the stomach has been associated with various gastric diseases: gastritis, peptic ulcer disease, gastric adenocarcinoma, and gastric mucosa-associated lymphoma [1]. CD4+ T helper cells (Th) are recognized as a key component of the adaptive immune response to extracellular bacteria and a dominant component of immune responses to *H. pylori* [2]–[5]. However, the mechanisms by which CD4+ T cells control *H. pylori* infection, disease and the associated gastric immunopathology are incompletely understood.

Th1 cells are induced by IL-18, IL-12 and IFNγ and express T-bet and STAT1 [6], which delineate their effector function. IFNγ secreted by Th1 cells activates effector functions of macrophages and dendritic cells (DC) in the gastric LP. IL-17-producing Th17 cells promote effector and inflammatory responses that can aid in fighting infections but can also be implicated in tissue damage. Their induction is determined by the combination of IL-6 and TGF-β in the tissue environment, which activate STAT3 and RORγt, two transcription factors involved in Th17 differentiation [7]. IL-17-producing cells enhance epithelial and neutrophil-derived antimicrobial activity and bacterial clearance during early infection with enteroaggregative *Escherichia coli* (EAEC) [8]. Th17 cells can also produce IL-22, which alone or in combination with IL-17 induces the production of antimicrobial peptides involved in bacterial clearance [9]. In contrast to Th17 cells, regulatory T cells (Tregs) are the main anti-inflammatory CD4+ T cell phenotype and their primary role is to down-modulate effector or inflammatory responses, thus facilitating mucosal homeostasis [10].

The genetic makeup of the host and its interaction with *H. pylori* predispose to clinical outcomes during infection [11]. The nuclear receptor peroxisome proliferator activated receptor gamma, (PPARγ) is a crucial regulator of immune responses [12]. We recently demonstrated that gastric colonization with *H. pylori* ameliorates glucose homeostasis in mice through a PPARγ-dependent mechanism involving the modulation of macrophage and Treg cell infiltration into the abdominal white adipose tissue and neuroendocrine changes in the stomach [13]. Interestingly, two recent clinical studies suggest an association between PPARγ and *H. pylori*-related gastric carcinoma [14], [15]. Also, PPARγ is upregulated during *H. pylori* infection [16], [17]. Furthermore, disruption of the PPARγ pathway by microRNA-146b may be implicated in the regulation of Th17 responses and colitis in *Clostridium difficile*-infected mice [18], and PPARγ tightly controls the plasticity of Th17 cells towards an iTreg phenotype [19]. Despite these advances in understanding the role of PPARγ in mucosal immunoregulation, the mechanisms underlying the modulation of gastric mucosal effector and regulatory pathways during *H. pylori* infection are not completely understood.

Results of human studies support the theory that pathogenic subsets of T cells are instrumental in inducing *H. pylori*-associated gastritis and ulcers [2], [20]. More, specifically, patients with peptic ulcer disease exhibit stronger Th1 and Th2 responses to *H. pylori* infection than asymptomatic carriers, whereas the latter exhibit a Treg-predominant response during infection [2], suggesting that Treg cells might contribute to the persistence of *H. pylori* in the stomach as a harmless commensal organism. Indeed, IL-10-producing Treg cells were particularly abundant in the gastric mucosa of healthy carriers compared to peptic ulcer disease patients [2]. Thus, CD4+ T cells play a decisive role in initiating and shaping the progression of disease and pathological outcomes in *H. pylori* infected individuals.

Mathematical modeling provides novel means of synthesizing cellular, molecular and tissue-level data into a common systems-level framework. Herein, we used two complementary types of modeling to study the impact of *H. pylori* infection in effector and regulatory pathways at the gastric mucosa. In ODE-based modeling, the variables of the equations represent average concentrations of the various components of the mathematical model whereas ABM takes into consideration the rules and mechanisms of behavior of the individual components of the system and spatiotemporal distribution of agents within the system. In contrast to ODE models that have fully developed, mature and automated systems of parameter estimation, a key limitation of ABM is that sensitivity analysis and parameter estimation methods are immature. To investigate how the interplay between CD4+ T cells and other immune and epithelial cell subsets in the gastric mucosa contributes to driving gastric pathology, we formalized a computational model of *H. pylori* infection using ODE and ABM approaches sequentially. We show that *H. pylori* infection triggers a predominant infectious dose-dependent Th1 response that is paralleled by a concurrent Treg response at the gastric mucosa. We also provide evidence in support of a role for increased effector T cell responses and the loss of PPARγ as key contributors to gastric immunopathology during *Helicobacter* infection. Furthermore, our simulations predict that the main cause of gastric damage in the chronic phase of the infection is the pro-inflammatory and effector immune response driven predominantly by effector T cells.

## Materials and Methods

### Ethics statement

All experimental protocols were approved by the Virginia Tech institutional animal care and use committee (IACUC) (Protocol Numbers: 10-087-VBI & 11-189-VBI) and met or exceeded guidelines of the National Institutes of Health Office of Laboratory Animal Welfare and Public Health Service policy. Animals were under strict monitoring throughout the duration of the project and all efforts were made to minimize unnecessary pain and distress. Mice were euthanized by carbon dioxide narcosis followed by secondary cervical dislocation.

### Computational modeling

**Figure 1:**
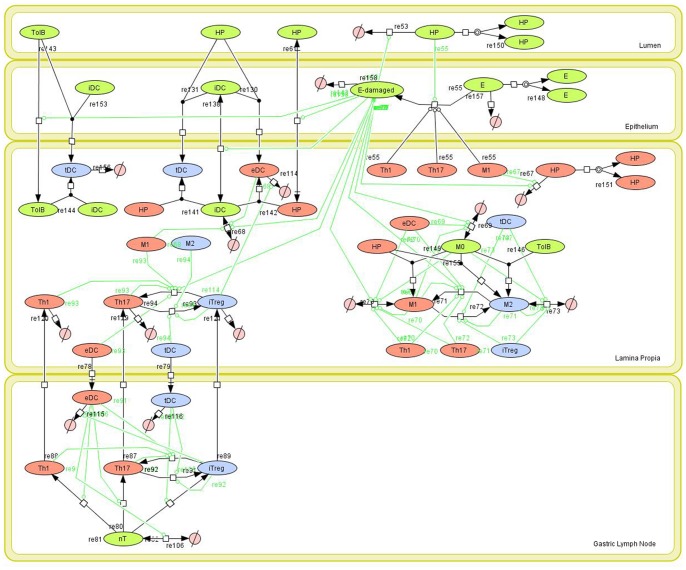
Network model of the mucosal immune responses during *Helicobacter pylori* infection. Systems Biology Markup Language (SBML)-compliant network of the interactions between *H. pylori* and cells participating in the innate and adaptive immune response such as macrophages (M1 and M2), dendritic cells (tDC and eDC), epithelial cells (E) and CD4+ T cell subsets (Th1, Th17, iTreg) in the gastric lumen, the epithelium, lamina propria (LP) and the gastric lymph nodes (GLN).

The computational model of the mucosal immune responses to *H. pylori* was developed in the following steps: first, the structure model as shown in Figure 1 was developed using CellDesigner, a widely used Systems Biology Markup Language (SBML)-compliant network structure model development tool. Immune responses to *H. pylori* represented in the model were based on a comprehensive and thorough literature review as well as time-course data generated by us. The inflammatory network portrayed is encoded as follows: Inflammation is initiated when *H. pylori* is innoculated in the gastric lumen. *H. pylori* lives in close proximity to the epithelial lining (i.e., floating on the mucus barrier) and can adhere directly to the host cell membrane and deliver toxins. The virulence factor CagA is injected directly into host cells by the bacteria through a type IV secretion system. CagA’s ability to perturb cell polarity is important for the efficient survival and growth of *H. pylori* on the apical surface of the host cell, therefore, being able to replicate in the lumen [21]. Epithelial cells in contact with *H. pylori* initiate a pro-inflammatory response characterized by production of chemokines, activation of DCs, macrophages and T cells [22]. *H. pylori* can also translocate and migrate into the gastric LP, thus attracting effector DCs [23], directing tolerogenic programming of DCs [24] and enhancing M1 polarization [25]. As expected, APCs engulfing *H. pylori* will display *H. pylori* antigenic determinants associated with MHC and will activate effector and regulatory CD4+ T cell differentiation. The regulation of DCs can restrict different phenotypes, such as Th1 [26]. The secretion of different factors such as IFNγ, IL-17 or IL-1β will activate macrophage differentiation [27] and these differentiated macrophages will help to clear *H. pylori* in the gastric LP. At the same time, *H. pylori*-infected macrophages can induce Th17 cell responses as a positive feedback loop [28]. During this literature search, a database used for model calibrations was also created. Secondly, we implemented the model in both COPASI and ENISI with dynamics of species and reactions defined. COPASI [29] is a widely used tool for ODE-based modeling; ENISI [30] a short name for Enteric Immunity Simulator, is an agent-based modeling tool, which has been developed by the Center for Modeling Immunity to Enteric Pathogens. The averaged-based ODE-based approach can provide mature and computationally efficient numerical algorithms especially for model calibration for modeling average behavior, while the agent-based approach can provide modeling capabilities of individual based behavior, stochasticity, and cell movements easily. The COPASI modeling tool can run in both local machines and condor clusters through an online job submission system. The agent-based modeling tool, ENISI, is high-performance computing (HPC)-based and it runs on our super computer system Shadowfax, a hybrid cluster with 912 processor cores, 5.4 TB of RAM, 40 Gb/s InfiniBand network and 80TB parallel storage. An online job submission system of ENISI has been developed for submitting *in silico* experiments through a web interface. These two approaches complement each other. Third, the model was calibrated using the calibration database including time-course data (Table S1) and assuming some biological facts regarding the behavior of specific cell types in the system (Table S2). A comprehensive list of markers used in flow cytometry to characterize immune cell populations can be found in Table S3.

Sensitivity analysis and parameter estimation have been performed in COPASI using a Particle Swarm algorithm and in ENISI using statistical data mining and variance-based analysis techniques, such as local sensitivity analysis using partial factorial experiments and sparse designs. In parallel with the computational modeling effort, we identified key experiments in mice to validate model predictions. The iterative computer modeling and experimentation cycle has provided a more complete systems-level understanding of the cellular mechanisms underlying immune responses to *H. pylori*. Novel ideas and hypotheses can be easily generated and tested in silico with significant time and cost savings. Therefore, the model was able to predict trends in the behavior of distinct cell types and these computational predictions were validated with experimental data (Table S4). The model developed in this study is published through the MIEP web portal at www.modelingimmunity.org and available at Biomodels.net (MODEL1307130000). More specifically, in the MIEP website, a tool called CellPublisher is used to publish the annotated model on the web portal that can allow users to navigate the network model in Google-map way and the annotations including the protein structure are presented as tags and 3-D animations.

### Mice and *H. pylori* Challenge

Eight to twelve week-old wild-type C57BL/6 mice were fasted for 8 hours and challenged via orogastric gavage with either PBS or 500 µL of 5×10^7^, 10^8^, 10^9^ or 10^10^ CFU/mL of *H. pylori* strains 26695, SS1 or PM-SS1, on days 0, 2 and 4. Urea was added to the drinking water at a concentration of 5% w/v to facilitate bacterial colonization. Mice were checked daily for signs of disease. At day 7, 14, 30 and 60 post infection mice were euthanized and spleen, gastric lymph nodes (GLN) as well as the stomach were excised for further analysis. Mice were housed under a 12∶12 light-dark ratio at the animal facilities at Virginia Tech. All experimental procedures were approved by the Institutional Animal Care and Use Committee of Virginia Tech and met or exceeded the requirements of the Public Health Service/National Institutes of Health, the Animal Welfare Act and Public Health Service policy. Animal experimentation was performed under IACUC protocols 10–087 VBI and 11–189 VBI.

### *H. pylori* culture

*H. pylori* strain 26695 (ATCC), SS1 and PM-SS1 were grown on plates prepared with Difco Columbia blood agar base (BD Biosciences) supplemented with 7% of horse blood (Lampire) and antibiotics at 37°C under microaerophilic conditions. To prepare whole cell (WC) bacterial antigens, bacteria were inactivated with 4% formaldehyde for 26 hours followed by two washing steps with PBS. Inactivated WC *H. pylori* antigen preparations were resuspended in PBS, quantified and stored at −20°C until further use. Bacterial inactivation was confirmed by culturing formaldehyde treated *H. pylori* for at least 4 days as described above.

### Preparation and processing of single cell suspensions

Spleens and GLN were excised and crushed in PBS/5% FBS using the frosted ends of two sterile microscope slides and a syringe plunger, respectively. Single cell suspensions were centrifuged at 300×g for 10 min and washed once with PBS. In case of splenocytes, red blood cells were removed by osmotic lysis prior to the washing step. For LPL isolation, stomachs were cut open and rinsed with PBS prior to 10 min treatment with 5% Acetylcysteine (Sigma) in HBSS with 2.5% Hepes and 10% FBS, at room temperature for 10 min. After treatment, stomachs were digested with 300 U/mL of collagenase (Sigma) and 50 U/mL of DNAse (Sigma) for 90 min at 37C under constant agitation and the cell suspension was filtered with a 100µm strainer. Single cell suspensions from GLN and spleen were either freshly stained for flow cytometry or stimulated with 5 µg/mL of plate-bound anti-mouse CD3 (BD Biosciences) for 6 hours. To inhibit protein secretion from cells, GolgiStopTM (BD Biosciences) was added for the last 4 hours of incubation according to the manufacturer’s instructions. Lymphocytes from LPL extraction were enriched using a 44/67% Percoll gradient, washed in PBS and resuspended in FACS buffer. Flow cytometric analysis of *ex vivo*-stimulated cells was performed to assess phenotype and function different immune cell populations.

### Immunophenotyping and cytokine analysis by flow cytometry

For fluorescent staining of immune cell subsets 4–6×10^5^ cells were incubated for 20 min with fluorochrome-conjugated primary mouse specific antibodies: anti-CD3 PE-Cy5 clone 145-2C11 (eBioscience), anti-CD4 PE-Cy7 clone GK1.5 (eBioscience), anti-CD4 APC clone RM4-5, anti-CD45 APC-eFluor780 clone 30-F11 (eBioscience) and anti-CD25 Biotin clone 7D4 (BD Biosciences). Cells were washed with FACS buffer (PBS supplemented with 5% FBS and 0.09% sodium azide) and incubated for another 20 min with PE-Texas Red-conjugated streptavidin (BD Biosciences). For intracellular staining of transcription factors and cytokines, cells were fixed and permeabilized using a commercial kit according to the manufacturer’s instructions (eBioscience). Briefly, cells were fixed and permeabilized for 20 minutes, Fc receptors were blocked with mouse anti-CD16/CD32 FcBlock (BD Biosciences) and cells were stained with fluorochrome-conjugated antibodies towards anti-mouse/human Tbet PerCP-Cy5.5 clone 4-B10, anti-mouse, FOXP3 FITC clone FJK-16s, IL-17A APC clone eBio17B7 and IFN-γ PE-Cy7 clone XMG1.2 (eBioscience). All samples were stored fixed at 4°C in the dark until acquisition on a LSR II flow cytometer (BD Biosciences). A live cell gate (FSC-A, SSC-A) was applied to all samples followed by single cell gating (FSC-H, FSC-W) before cells were analyzed for the expression of specific markers. Data analysis was performed with FACS Diva (BD Biosciences) and Flow Jo (Tree Star Inc.).

### Statistical analysis

Parametric data were analyzed by using the ANOVA followed by Scheffe’s multiple comparison method. Nonparametric data were analyzed by using the Mann-Whitney’s *U* test followed by a Dunn’s multiple comparisons test. The ANOVA was performed by using the general linear model procedure of SAS, release 6.0.3 (SAS Institute). Statistical significance was assessed at a *p-value* ≤0.05. To assess the significance in the knock-out models when compared to the wild-type we used a functional T-test approach. The objective of the functional T-test is to evaluate whether two groups of functional curves are statistically different. Specifically, we defined 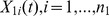 as the functional curves of the first group and 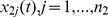 as the function curves for the second. To evaluate the difference between two groups of curves, we considered the absolute value of a t-statistic at each point:
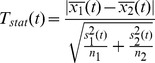
Where 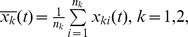 and 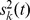 is the sample variance of 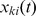 at the time point 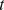 The values of 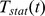 can provide the relative difference of the two groups of curves, For the test statistic, we considered the maximum value of 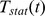. To find a critical value of this statistic, we use a permutation test by first performing a randomly shuffle of the labels of the curves and then recalculating the maximum of 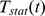 with the new labels. Repeating this procedure many times can provide an empirical null distribution of 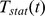. Therefore, we can calculate the critical point as a reference for evaluating the values of observed 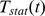. The values over the calculated threshold will be viewed as statistically significant. To evaluate the effect of epithelial cell damage in the ABM model, preliminary analysis for model sensitivity was conducted. Specifically, we collected the values for number of cells that modulate epithelial cell damage from the ABM model output datasets and grouped them by genotypes. The maximum value of cell counts was used to make 10 cell count range uniformly. Such range was then used to construct heat maps showing the relative significance of different immune subsets over the epithelial cell damage.

## Results

### Mathematical modeling of mucosal immune responses to *H. pylori* infection

Given the complexity, nonlinearity and abundance of feedback loops in mucosal immune responses to *H. pylori* and to facilitate a better understanding of the mechanisms underlying such immune responses at the systems level, we constructed a SBML network model depicting the major effector and regulatory pathways evoked during *H. pylori* infection [31] (Figure 1). The model is comprised of four distinct compartments: the lumen, epithelium, gastric LP, and gastric lymph nodes (GLN). The same network was used for ODE and ABM efforts. The ODE model is comprised of 24 species and 43 reactions in both gastric mucosa and GLN, and encompasses immune networks, which lead to 19 ordinary ODE (Figure S1). In both ABM and ODE models, effector cell types such as M1 macrophages, Th1, Th17, and pro-inflammatory epithelial cells secrete cytokines and chemokines that i) recruit immune cells, ii) promote activation and differentiation to inflammatory phenotypes, and iii) secrete effector molecules that destroy bacteria and may cause tissue damage. Regulatory hematopoietic cells such as M2 macrophages, tolerogenic DCs, and Treg cells act antagonistically to their inflammatory/effector counterparts through various contact- and cytokine-dependent mechanisms [32]–[34].

Immune cell populations are categorized by immunological state (resting, active inflammatory, regulatory), epithelial cells are sub-divided in healthy and damaged subtypes. All populations are further compartmentalized by location in one of four tissue sites (GLN, gastric LP, epithelium and lumen). Computational variables are the absolute number of cells in each compartment over time. Cell differentiation is represented as a flow from one cell-type to another, and migration as a flow from one location compartment to another. In the ABM, individual cells are represented as state-defined agents with concrete spatiotemporal features that follow the model paradigm, changing their state and triggering different reactions as time progresses. This set of agents encapsulates the behaviors of the various individuals that form the system and execution consists of emulating these behaviors after *H. pylori* infection.

Our ABM represents the migration of *H. pylori* from the mucous layer of the gastric lumen towards the epithelium and invasion of the LP. However, upon contact of the bacterium with a healthy epithelial cell, represented as E, bacterial infection is initiated and this epithelial cell starts secreting inflammatory mediators, represented as E_damaged in the network model, thus triggering an inflammatory response that affects macrophages and DCs locally in the LP, which can adopt effector (M1 and effector dendritic cell or eDC) or regulatory (M2 and tolerogenic dendritic cell or tDC) phenotypes. Tolerogenic bacteria (TolB) are also represented, highlighting how commensalism helps to maintain a regulatory phenotype at the gut mucosa.

The ODE model was calibrated using a Particle Swarm algorithm [35] implemented in COPASI [36] with *in vivo* flow cytometry data (Table S1). Calibration datasets were obtained by intragastrically infecting C57BL/6 mice with *H. pylori* strain 26695 and assaying immune cell subsets (Table S3) in the stomach and gastric lymph nodes (GLN) on days 0, 7, 14, 30 and 60 post-infection. In the case of the ABM model, ODE-based model parameter values were used to provide initial values and to narrow the search for parameter values in the estimation. Since parameter estimation techniques in stochastic agent-based processes are not as mature as in ODE tools, starting the parameter value search near the ODE solution facilitates the subsequent trial-error experimentation to find the right parameter that will represent best the experimental data. For this reason, we use ODE-based parameters as a first step in the parameterization process in the ABM-based model. This parameter evolution from the initial ODE values to the final ABM parameter set is represented in Table S5.

### *H. pylori* modulates CD4+ T cell subsets in the GLN and gastric LP

**Figure 2:**
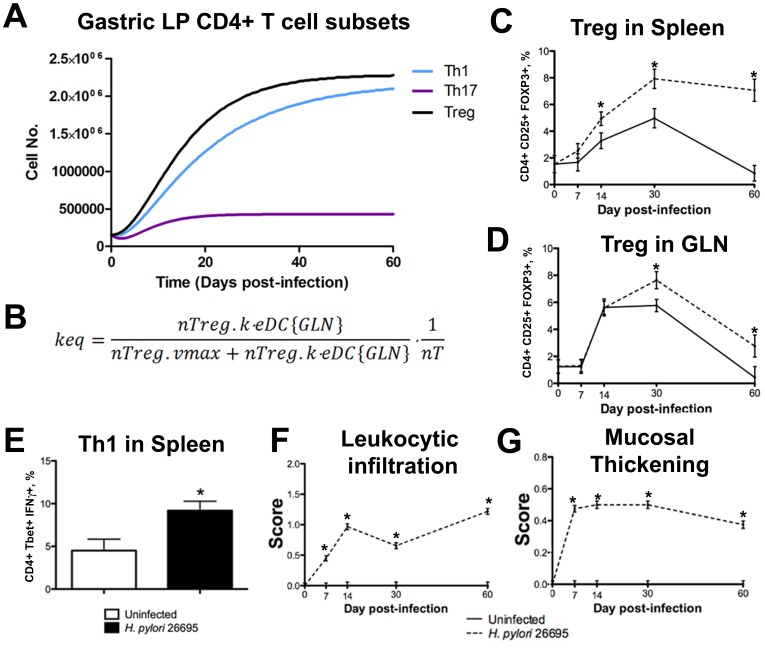
Effector and regulatory CD4+ T cell subsets modulate the immune responses during *Helicobacter pylori* infection. (A) *In silico* time-course experiment performed with a challenge of 5×10^7^ colony forming units of initial *H. pylori* injected in the mathematical model, showing differences in numbers of gastric lamina propria (LP) CD4+ T cell subsets over time. (B) Equilibrium constant regulating CD4+ T cell gastric lymph nodes (GLN) differentiation in our computational model. (C, D) Flow cytometry analysis results showing differences in the percentages of regulatory T (Treg) cells in spleen and GLN. (E) Flow cytometric analysis showing differences in the expression of IFNγ+ Tbet+ CD4+ T (Th1) cells in the spleen at day 30 post-infection. (F, G) Histopathological analysis on the gastric mucosa showed lesions consistent with *H. pylori* infection. Mouse stomachs had increased leukocyte infiltration in the LP and gastric mucosal thickening due to epithelial cell proliferation.

Since CD4+ T cells play a crucial role in determining the outcome of disease during *H. pylori* infection, we sought to determine the relative contributions of effector and regulatory CD4+ T cell subsets in the gastric mucosa during infection. Our ODE modeling approaches showed a distinct time-dependent behavior in Th1, Th17 and iTreg cells represented in the mathematical model during *H. pylori* infection. Th17 cells slightly increased at day 10 post-infection, but as time progressed, they arrived to a plateau at lower levels than Th1 and iTreg cells. In contrast, iTreg cells increased, reaching a stable steady state around day 35 that persisted until day 60 post-infection (Figure 2A). Th1 cells chronically populated the gastric LP throughout the infection period, thereby contributing to the overall inflammation of the gastric LP. T cell responses at the gut mucosa were partially controlled by the balance between effector and tolerogenic DCs (eDC and tDC respectively) and the equilibrium constants in our computational model (Figure 2B). Flow cytometry analysis of tissues recovered from C57BL/6 wild-type mice infected with *H. pylori* strain 26695 demonstrated that Treg cells were present in both spleen (Figure 2C) and GLN (Figure 2D), and their numbers were increased starting at day 7 post-infection, reaching a peak around day 30 and persisting throughout the entire infection period. Moreover, we observed the presence of significantly increased percentages of Th1 cells in spleens of *H. pylori*-infected mice on day 30 post-infection (Figure 2E). Histopathological analysis of gastric specimens showed mild leukocytic infiltration on the gastric LP (Figure 2F) and a slight but significant increase of gastric mucosal hyperplasia (Figure 2G). Additionally, increasing infectious doses of *H. pylori* innoculation elicited a dose-response behavior for Th1 (Figure S2A) and Th17 cells in the gastric LP (Figure S2B). To validate this model prediction, we performed a dose-response study where mice were inoculated with 0, 10^8^, 10^9^ or 10^10^ CFU/mL *H. pylori* strain 26695. *In vivo* results demonstrated that the expression of T-bet and RORγt, as well as the production of IFNγ, all within the CD4+ T cell compartment, is dependent on the initial inoculation dose of *H. pylori* (Figure S2C-S2E).

### Myeloid cell-specific PPARγ deletion modulates macrophage, dendritic cell, and T cell differentiation during *H. pylori* infection

**Figure 3:**
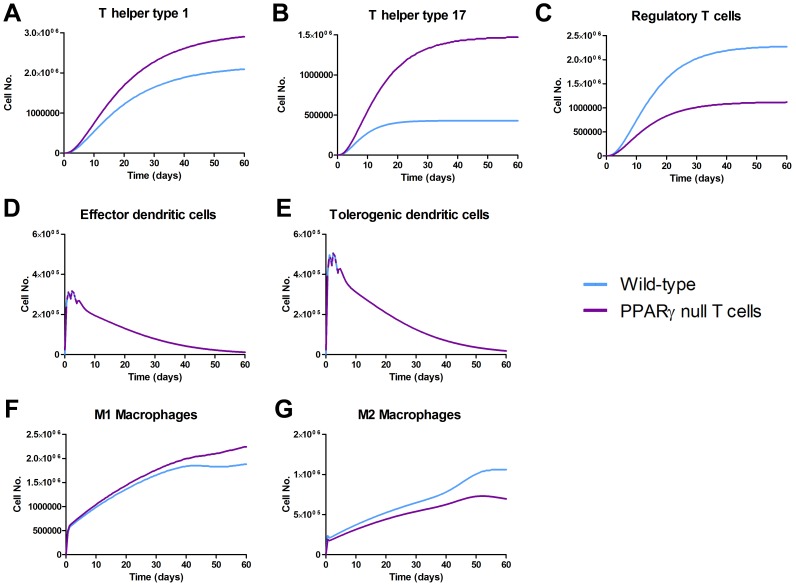
*In silico* dynamics of gastric mucosal T cell subset in wild-type and T cell-specific peroxisome proliferator-activated receptor γ (PPARγ) knockout mice following infection with *Helicobacter pylori*. Time-course experiments following infection with 5×10^7^ colony-forming units (CFU) of *H. pylori* to determine dynamics on CD4+ T cell phenotypes. Blue lines represent wild type mice and violet lines represent T cell-specific PPARγ knockout mice. T helper (Th) 1 (A), Th17 (B), induced regulatory T cell (iTreg) (C), effector dendritic cells (D), tolerogenic dendritic cells (E), M1 macrophages (F) and M2 macrophages (G) are illustrated.

PPARγ is recognized as an important immunoregulatory molecule in the gastrointestinal mucosa. To elucidate the role of PPARγ in both myeloid and T cell subsets during *H. pylori* infection, we created cell-specific knockout models. First, to simulate the myeloid-specific PPARγ knockout system we reduced the maximum rate of undifferentiated macrophage M0 transitioning to alternatively-activated M2 macrophages, reduced the maximum rate of M1 conversion to M2 macrophages [37], [38], and reduced the rate of iDC switching to tDC by cytokines [39]. In the case of the T cell-specific PPARγ knockout, we lowered the rate of naïve CD4+ T cells becoming iTreg [40], the maximum rate of Th17 differentiation to iTreg [38] and the rate of constitutive iTreg stimulation [40], [41]. Simulation results showed a marked impact of the loss of PPARγ on myeloid cell populations following infection. Specifically, when compared to the wild type model, there was an increase in eDC and decreased tDC in both the gastric LP and the GLN (Figure S3A and S3B). Similarly, elevated M1 and reduced M2 macrophage numbers in the LP were observed (Figure S3C and S3D). Along with the elevated inflammatory response in the myeloid cell populations we found a decline in *H. pylori* in the gastric lumen indicating more efficient clearance (Figure S3E) and a slightly elevated epithelial cell death (Figure S3F) in the myeloid-specific PPARγ knockout model when compared to the wild-type system. The T cell-specific PPAR γ knockout model showed elevated Th1 and Th17 (Figure 3A and 3B) when compared to the wild-type model, whereas iTreg cell levels in the LP were dramatically decreased (Figure 3C). Interestingly, no differences in the numbers of effector or tolerogenic DC were observed (Figure 3D and 3E). However, a lack of PPARγ in T cells had a mild effect on macrophage populations, increasing the expansion of M1 macrophages (Figure 3F) and decreasing the numbers in the M2 alternatively activated macrophage subset (Figure 3G).

### Modeling stochasticity in cellular responses during *H. pylori* infection by using ENteric immunity sImulator (ENISI)

**Figure 4:**
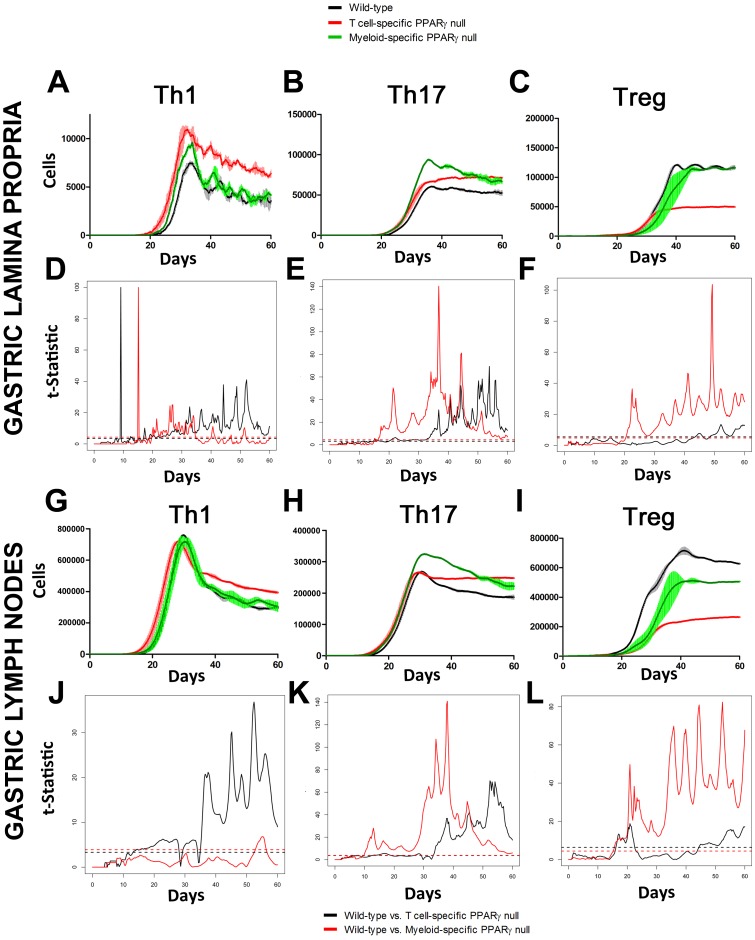
Enteric Immunity Simulator (ENISI) output results and assessment of the role of the Peroxisome Proliferator Activated Receptor γ (PPARγ) in both the myeloid and T cell subset modulated T cell responses after *Helicobacter pylori* infection *in silico* in the gastric lamina propria (LP) and gastric lymph nodes (GLN). The *H. pylori* ABM was run as a time-course for 60 days. Model parameters were changed to simulate myeloid or T cell-specific PPARγ knockout systems as described in Table S2. Dynamical variation of Th1 (Figure 6A, 6G) as well as Th17 (Figure 6B, 6H) and regulatory T cells (Figure 6C, 6I) changing over time were plotted. A functional T-test was used with 95% confidence interval to create statistics assessing differences in the myeloid and T cell specific PPARγ knock-out for Th1 (Figure 6D, 6J), Th17 (Figure 6E, 6K) and Treg (Figure 6F, 6L). A threshold value representing the critical value of significance vertically divides the plot into two parts, showing significant differences above the threshold. Data were obtained in 15 runs of the simulation for each different genotype.

To further characterize the immunological mechanisms underlying mucosal immune responses to *H. pylori* in a stochastic system, we used ABM based on parameter values derived from refinement on our initial ODE model parameters (Table S5). When probabilistic approaches are used, the complex immunological processes can be better represented. We adopted the ABM tool ENteric Immune Simulator (ENISI) developed by us and available at www.modelingimmunity.org [30]. To calibrate the ABM we used a set of parameters derived from our ODE-based modeling approaches as initial values before refinement. After implementing the model specification as well as the initial concentrations as previously described [42], we ran simulations up to 60 days post-infection and analyzed the changes in cell concentrations in both LP and GLN. Results expressed in heat map concentrations show a significant increase in the concentration of CD4+ T cells in both the gastric LP and the GLN (Figure S4). Taking a closer look at CD4+ T cell subsets following infection in the wild-type model, we observed that in the GLN, Th1 cells peaked on day 30 post infection and remained at high levels with fairly constant values throughout the rest of the infection period (Figure 4A, 4G). Th17 responses were induced in the GLN and later detected in the LP, together with a Treg cell response that persisted over time in both gastric LP (Figure 4B, 4C) and GLN (Figure 4H, 4I).

### ABM highlights the immunoregulatory role of PPARγ in modulating host responses towards *H. pylori in silico*

In order to investigate the role of PPARγ in mucosal immune responses to *H. pylori in silico*, we engineered T cell-specific and myeloid cell specific PPARγ knockout models. Specifically, to create an *in silico* cell-specific knockout model, rates of regulatory phenotype differentiation were lowered and rates controlling effector response in both LP and GLN were increased. A side-by-side comparison on the parameter changes implemented in the T cell- and myeloid cell-specific PPARγ knockout systems can be found in Table S6. We used a functional T-test to visualize statistically significant differences over time when comparing wild-type and the knockout models. Our results showed a statistically significant expansion of Th1 in the T cell-specific PPARγ null system when compared to the wild-type starting around day 35 and increasing the difference throughout the infection up to day 60 in the gastric LP (Figure 4A and 4D). In the GLN, significant differences were first detected around day 50 and peaked throughout the rest of infection (Figure 4G and 4J). No statistically significant differences in Th1 cell numbers were found between the wild-type and the myeloid cell-specific PPARγ knockout system (Figure 4A, 4D, 4G and 4J). Regarding Th17 cells, these simulations depicted the immunoregulatory role of PPARγ in the myeloid subset since we observed significant differences in enhanced Th17 responses in the myeloid cell-specific PPARγ knockout model when compared to the wild-type. More specifically, there was a statistically significant effect starting at day 30 and remaining significant until day 60 in the gastric LP (4B and 4E) and the GLN (4H and 4K). Th17 cell numbers were also significantly higher in the T cell-specific PPARγ knockout model when compared to the wild-type model in both LP (Figure 4B and 4E) and the GLN (Figure 4H and 4K). T cell-specific PPARγ deficiency significantly impaired the expansion of the iTreg cell compartment starting at day 30 and showed an oscillatory behavior and significant differences until day 60 in the gastric LP (Figure 4C and 4F). In the GLN, the differences between the T cell specific PPARγ knockout and the wild-type were significantly noticeable during the whole period of infection (Figure 4I and 4L). The myeloid cell-specific PPARγ knockout showed similar differences. In the case of the GLN, the myeloid cell-specific PPARγ knockout model showed significant differences when compared to the wild type up to day 30 post-infection (Figure 4I and 4L).

### Gastric histopathological lesions are formed as a consequence of effector immune activation during the chronic phase of the *H. pylori* infection

**Figure 5:**
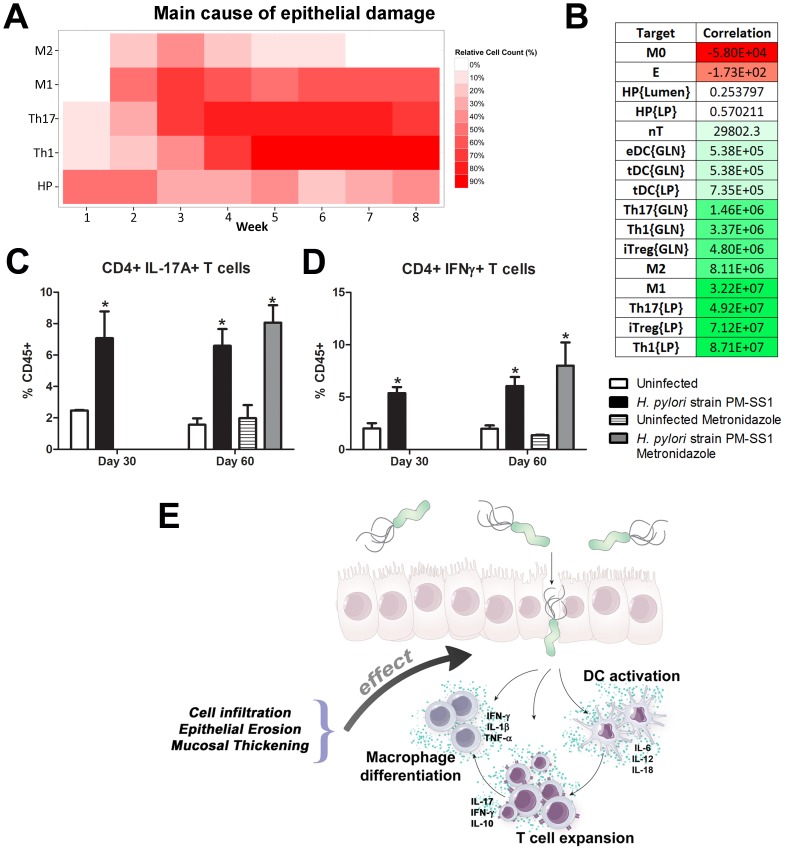
Sensitivity analysis of factors involved in gastric inflammatory lesion formation following *Helicobacter pylori* infection. Healthy epithelial cells changing state into pro-inflammatory epithelial cells, thereby contributing to the formation of gastric lesions. (A) Differential time-dependent patterns of lesion formation in the early, meridian and chronic-late stage of infection. (B) ODE-based deterministic sensitivity analysis on pro-inflammatory epithelial cells, as variables, and its formation at day 60 post-infection using a delta factor of 0.001 with a delta minimum of 1×10^−12^. (C) Flow cytometric analysis showing differences in the expression of CD4+ IL-17A+ cells in the gastric lamina propria after *H. pylori* infection. (D) Flow cytometric analysis showing differences in the expression of CD4+ IFNγ+ cells in the gastric lamina propria after *H. pylori* infection. (E) Cartoon model representation of the effect of DC activation, T cell expansion and macrophage differentiation on the formation of histopathological lesions in the gastric lamina propria (LP) during *H. pylori* infection.

**Figure 6:**
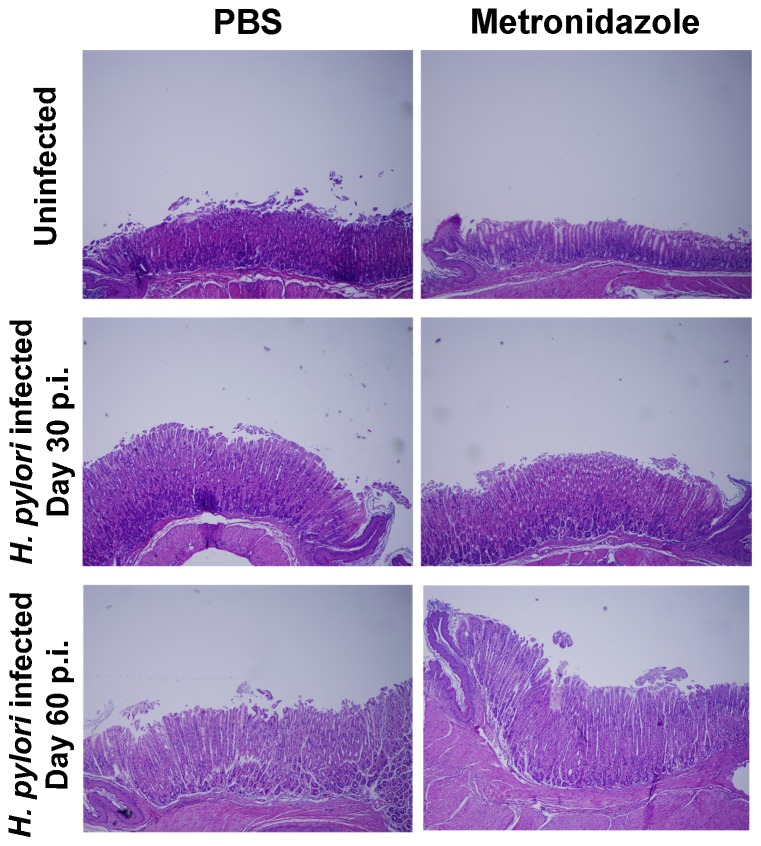
Histopathological assessment of the gastric mucosa of mice after *Helicobacter pylori* infection. Representative photomicrographs of stomachs from either non infected or *H. pylori*-infected mice following administration of PBS or metronidazole treatment. Original magnification 40×.

Sensitivity analysis shows whether the model predictions are sensitive to model parameters and concentrations. It also helps to understand complex relationships between distinct model variables. We extended our modeling approaches to determine which are the main factors involved in gastric lesion development during *H. pylori* infection by using sensitivity analysis. Our sensitivity analysis results using ABM showed how at the early stage of infection (up to week 2 post-challenge), the epithelial cell damage is mainly caused by the bacterium (Figure 5A). Interestingly, as the infection progresses, we observed a trend towards Th1 and Th17 cells triggering epithelial cell damage starting 3 weeks post-infection. At the chronic phase of the infection (i.e., around 6–8 weeks post-infection), our results showed a dominant role of Th1 and Th17 effector cells in inducing epithelial cell damage (Figure 5A). *H. pylori* induced epithelial cell damage throughout the infection. However, at a later infection stage, the induction of damaged epithelial cells by the effector Th1 and Th17 phenotypes overshadowed the effect of *H. pylori* itself. Of note, sensitivity analysis performed in the deterministic model at day 60 post-infection also showed how Th1 and Th17 cells in both LP and GLN were contributing to the epithelial cell damage as well as M1 macrophage differentiation, whereas *H. pylori* had a more limited impact on the formation of such lesions (Figure 5B). This sensitivity analysis data suggested that the main contributors of histopathological damage in the gastric mucosa at a chronic stage of infection are Th1 and Th17 effector responses. Going one step further from the model prediction, we hypothesized that the effector T cell response and not the bacterium itself is the main cause of epithelial cell damage during the chronic phase of *H. pylori* infection. To validate this hypothesis, C57BL/6 wild-type mice were infected with *H. pylori* strain PM-SS1 to characterize mucosal immune responses and to assess contributors to epithelial cell damage. In this study, a group of mice was treated with metronidazole, an antibiotic shown to effectively clear *H. pylori* from the stomach [43]. This experimental design (Figure S5) allows us to begin dissecting the effects of the dynamics of the host response versus the bacterium in the chronic phase of disease. On day 30 post-infection, a group of mice were euthanized for baseline immunological measurements and the rest were divided into two groups: one treated with metronidazole and one treated with sterile PBS as a control. At day 60 post-infection the remaining mice were euthanized for histological and immunological analyses. Immunophenotyping results showed a pronounced increase of IL-17A- (Figure 5C) and IFNγ-producing cells (Figure 5D) in the gastric LP after 30 and 60 days post-infection. Metronidazole treatment did not affect effector cytokine expression. These results suggested that effector T cell responses are implicated in lesion development during infection as showed in a cartoon model representation, highlighting the involvement of DC, T cells and macrophages on the formation of gastric lesions in the LP is shown in Figure 5E. To determine the presence of gastric mucosal lesions we examined H&E-stained gastric samples. The results show an increased mucosal thickness and mild infiltration of inflammatory cells, which were more accentuated on day 60 compared to day 30 post-challenge (Figure 6). At day 60, no differences were found between mice that received antimicrobial therapy to eradicate *H. pylori* and those that remained untreated. In both groups, we observed a significant increase in the thickness of the gastric mucosa characterized by the elongation of the gastric pits and moderate depletion of chief cells at the base of glands (Figure 6).

## Discussion

*Helicobacter pylori* infection is associated with an increased risk for developing gastric and duodenal ulcers, gastric mucosa-associated lymphoid tissue lymphoma and gastric adenocarcinoma [44]–[46]. There is also increasing evidence of *H. pylori* providing protection against esophageal and cardial pathologies [47]–[50], childhood asthma [51]–[53], childhood allergies [52], [54], obesity and diabetes [13]. The immunological mechanisms underlying this protective effect of *H. pylori* acting as a commensal bacterium versus a pathogenic organism are incompletely understood.

Here, we combined computational modeling and animal experimentation approaches in an iterative cycle aimed at investigating immunological mechanisms underlying the modulation of mucosal immune responses to *H. pylori*. Overall, our modeling results demonstrate that CD4+ T cells are implicated in *H. pylori* clearance from the gastric LP. Previous studies have shown that *H. pylori*-specific CD4+ T cells preferentially home and accumulate in the infected stomach and L-selectin, C-C chemokine receptor type 4 (CCR4) and macrophages derived chemokines (MDC) play a critical role in Th cell recruitment and trafficking [20]. Indeed, our immunophenotyping results show an increased expression of CD4+ T cell specific IL-17A and IFNγ in the gastric LP following *H. pylori* infection.

Chronologically, we have demonstrated that *H. pylori* evokes a weaker Th17 response, followed by a dominant and more persistent Th1 response that is paralleled by an immunoregulatory CD4+ T cell response characterized by Treg cells slowly accumulating at the beginning of the infection, reaching the highest levels at 30 days post-infection that is sustained over time. Our *in vivo* data matches the simulation results in splenic and GLN Treg cells. Not surprisingly, Treg cell responses suppress inflammation and ulceration caused by the excessive host response to the bacterium [46], [55] and their balance with other subsets is critical for preventing gastric and duodenal ulcers [56]. Treg cell numbers were significantly decreased in the GLN at day 60, suggesting a potential migration from inductive to effector mucosal sites. We have also observed a downregulation of Th17 responses *in silico* after the first stage of infection, suggesting that effector Th1 cells might be implicated in the chronicity of infection and mucosal lesion development. The plasticity between Th17 and Treg cells, and the mechanisms controlling such phenotypic re-programming are under investigation. Interestingly, pro-inflammatory Th17 cells can acquire a regulatory phenotype with *in vivo* immunosuppresive properties [57]. We recently characterized PPARγ as a key modulator of Th17 plasticity towards an iTreg phenotype by using a combination of systems modeling of CD4+ T cell signaling and *in vivo* validation [41]. Gastric mucosal IL-17-producing cells can also contribute to development of gastric lesions [58], [59]. IL-17 also plays an important role in promoting B cell crosstalk and decreasing inflammation, therefore accentuating regulatory responses [60]. Hence, Th17 cells can exert dual functions as effectors of pathogenic tissue-damaging responses, but also as immunoregulatory responses driven by the secretion of IL-17 and IL-22 [61].

CD8+ T cells are crucial in *H. pylori* infection in humans and pigs [62], [63]. However, our experimental data in mice did not show any differential behavior in CD8+ T cells. Therefore, given the focus of our experimental questions on CD4+ T cells and our initial CD8+ T cell data we decided not to include CD8+ T cells and focus modeling work in the potential involvement of Th1 and Th17 effector responses in the induction of gastric lesions during the chronic phase of *H. pylori* infection. Of note, our sensitivity analysis highlighted the important role of Th1 and Th17 effector cells in the induction of gastric mucosal lesions in the chronic phase of the infection, even overshadowing the role of *H. pylori* itself. Consistent with our computational simulations, our in vivo studies validated the hypothesis that the role of *H. pylori* in tissue damage during the chronic phase of the infection is dramatically reduced.

To study the mucosal immune responses to *H. pylori* at the systems level locally in the gastric mucosa, we used ODE and ABM sequentially. First, our deterministic ODE model shed some new light on CD4+ T cell distribution after infection as well as the role of PPARγ during infection. Secondly, and because strategies for parameter estimation are neither fully developed nor automated in ABM, the ODE model provided a set of parameter values that were after used as a starting point for our ABM modeling. Since the ABM model uses a probabilistic approach, in comparison to the constant-based ODE model, further refinement on the parameter values was needed and trial-error simulations were performed. Despite of this additional step, having an initial parameter range based on the ODE model has narrowed the range and improved efficiency in the parameter estimation process for ABM. Furthermore, ABM adds randomness to the biological systems, which can help to better represent complex cellular responses and to take into account the individual and emerging behaviors of cells as well as the role spatiotemporal features. Thus, stochastic models can provide novel insights into the effect of cognate and non-cognate interactions, representing entire systems with a greater granularity and capturing cell-cell interactions. By simulating individual behaviors of agents, ABM better represents cross-linked, complex and nonlinear processes with multiple feedback loops and, provides a more comprehensive and interactive modeling of mucosal immune responses to *H. pylori*. The ability of ABM to encompass multiple scales of biological processes and incorporate spatiotemporal considerations, coupled with an intuitive modeling paradigm, underscores the added value of this modeling framework in translational systems immunology and immunoinformatics research. Our combined modeling ODE and ABM approaches provided evidence suggesting that the cause for gastric lesions during the chronic stage of the infection were effector Th1 and Th17 cell subsets as well as inflammatory macrophages. Furthermore, both ODE and ABM based models could generate several predictions that were validated *in vivo*, such as detecting *H. pylori* dose-response dynamics in Th1 and Th17 (Figure S3), showcasing the predictive power of the *H. pylori* model. Future studies will investigate the spatiotemporal progression of lesions in relation to immune cell trafficking in the tissue space.

Instructive and selective factors play important roles in shaping the immune responses in the gut. CD4+ T cell and macrophage differentiation highly depends on the cytokine environment, an instructive factor, and the competition of cells for phenotype-changing cytokines (i.e., selective factors). However, secretion of cytokines and chemokines into the tissue environment by cells depends upon intracellular signaling pathways. Multi-scale modeling approaches that combine intracellular signaling pathways and tissue level modeling of cell-cell, cell-bacteria, and cell-molecule interactions will be necessary to fully represent the mechanisms underlying the actions of selective and instructive factors in mucosal immune responses.

PPARγ is a transcription that tightly controls many aspects of mucosal immune responses. For instance, PPARγ is a negative regulator of macrophage activation [64] and its inhibition contributes to systemic inflammation [65]. Myeloid cell-specific loss of PPARγ has been reported to enhance chemokine and adhesion molecule expression leading to improve recruitment of inflammatory Ly6C^hi^ monocytes to sites of inflammation and infection [66]. Indeed, our results show that PPARγ modulated effector and regulatory responses also during *H. pylori* infection. With the ability to create computational models and extensive *in silico* knockout systems, where expression of the molecule of interest is ablated, we demonstrated a suppression of M2 macrophages and tolerogenic DC, and an increase of M1 macrophages and effector DC in our myeloid cell-specific PPARγ knockout model. A decline in *H. pylori* in the lumen was also observed in the knockout model, indicating more efficient bacterial clearance in the PPARγ knockout model. This coincides with elevated epithelial cell damage, indicating that *H. pylori* could be removed at the expense of elevated gastric lesions. Thus, our results demonstrate that PPARγ in the myeloid subset has a major role in modulating and controlling pro-inflammatory versus anti-inflammatory cell profile and consequently, a central role on bacterial clearance. These findings are in line with a recent report indicating that the lack of PPARγ in myeloid cells confers resistance to *Listeria monocytogenes* infection [66], suggesting that a regulatory network in myeloid cells that is governed by PPARγ restrains bacteriocidal activity and recruitment of inflammatory/effector cell subsets to the mucosal sites.

In CD4+ T cells, PPARγ partially drives differentiation and plasticity between phenotypes [38], [41], [67]–[70]. Our *in silico* knockout studies in CD4+ T cells highlight the anti-inflammatory properties of PPARγ by observing a suppression of Treg cell numbers and enhancement of effector phenotypes such as Th1 and Th17 in the knockout when compared to the wild-type models. Interestingly, our simulations with the myeloid cell-specific PPARγ knockout system promoted Th17 differentiation and suppressed Treg expansion. Considering that CD4+ T cells have a pivotal role and crosstalk with different subsets of DC, our findings are in line with other studies suggesting that DC subsets affect Th17 differentiation and plasticity in humans, where CD14+ HLA-DR−/low myeloid derived suppressor cells (MDSC) induced FOXP3+ regulatory T cells whereas CD14+ HLA-DR+ MDSCs promoted the generation of IL-17 secreting CD4+ T cells [71]. Furthermore, myeloid APCs are essential for the induction of IL-17A+ FOXP3+ T cells from memory CCR6+ T cells or Treg cells [72]. These results point out for the first time that PPARγ in myeloid cells plays a central role in Th17 differentiation. In addition, the deletion of PPARγ in T cells had a milder effect in the expression of differentiated macrophages, increasing the numbers of the M1 population and decreasing M2 macrophages. These findings are in line with the importance between innate and adaptive immunity and how CD4+ T cell-derived cytokines can affect the differentiation into pro- versus anti-inflammatory phenotypes. Therefore, CD4+ T cells are necessary and sufficient for gastritis induction in the *H. pylori* infection model. We also observed an oscillatory behavior in the wild type Th1 cell population as well as the knock-out models in the gastric LP using our ABM approach. This phenomena observed in the continuum of interest may be related to biological feedback loops in mucosal immune responses that contribute to maintain homeostasis and priming. One possible explanation could be the iterative process by which dendritic cells enter the GLN and expose the antigen to CD4+ T cells, incrementing its concentration as the subpopulation expands, and decreasing it when chemotactic strategies make CD4+ T cells to leave the lymphatic compartment. Of note, our modeling work highlights CD4+ T cell priming in the GLN. Some studies also point that CD4+ T cells are also likely primers with *H. pylori* antigens captured in the small intestines, where the coccoid form of *H. pylori* is taken up by DCs in the Peyer’s Patches [73]. Future studies using multi-scale modeling will elucidate the relationship between the intracellular differentiation pathways, the link to different subsets in the innate immune system and the potential relationships that can rise oscillatory trends in the system.

In summary, we combined computational modeling approaches and mouse challenge studies to investigate how CD4+ T cells and other immune cell subsets are distributed in the gut mucosa during *H. pylori* infection. Our model simulated T cell responses to *H. pylori* by using both platforms: ODE and ABM. Our modeling efforts predicted higher levels of effector responses in both the LP and the GLN when deleting PPARγ, thus highlighting the role of PPARγ activation as a potential mechanism for modulating CD4+ T cell responses during bacterial infection and positioning PPARγ as a candidate for immunotherapeutics development. Future studies will more fully realize the potential of multiscale modeling to understand mucosal immunity.

## Supporting Information

Figure S1 **Ordinary Differential Equations (ODE) triggering activation and inhibition regulatory and effector pathways in our** ***H. pylori*** **infection model.** Briefly, mass action and contact dependent functions were used to reproduce *H. pylori* infection cell behaviors *in silico* based on the addition of *H. pylori* in the gastric lumen.
(TIF)

Figure S2 **T helper (Th)1 and Th17 responses during** ***Helicobacter pylori*** **infection are dose-dependent.** Computational simulations with the *H. pylori* model demonstrating a dose-response effect with the initial dose of *H. pylori* in (A) Th1 and (B) Th17. *In vivo* experimentation validating this prediction by observing increased levels of splenic (A) T-bet, (B) IFNγ and (C) RORγt with increasing concentrations in the initial dose of *H. pylori*.
(TIF)

Figure S3 **Predicted dynamics of gastric mucosal cell subsets and luminal** ***Helicobacter pylori*** **counts following** ***in silico*** **infection of wild-type and myeloid-specific peroxisome proliferator-activated receptor γ (PPARγ) knockout models.** Time-courses were run *in silico* with infections using 5×10^7^ colony forming units (CFU) of *H. pylori* to determine myeloid subsets dynamics. The blue lines represent the wild-type model whereas violet lines represent the PPARγ knockout model in (A) gastric lamina propria (LP) effector dendritic cells, (B) LP tolerogenic dendritic cells, (C) LP M1 macrophages, (D) LP M2 macrophages, (E) *H. pylori* loads in the stomach lumen and (F) epithelial cell damage following infection with *H. pylori.*
(TIF)

Figure S4 **Enteric Immunity Simulator (ENISI) output results after** ***Helicobacter pylori*** **infection** ***in silico*** **.** The *H.pylori*-implemented agent-based model was run as a time-course for 60 days. Heatmap representation of cell concentrations being modulated over time in the gastric Lamina Propria (LPL) and in the Gastric Lymph Nodes (GLN). Cell types are grouped by function and effector vs. regulatory mechanisms.
(TIF)

Figure S5 **Experimental design to validate model prediction on main inducers of histopathologcal changes during** ***Helicobacter pylori*** **infection.** Wild-type mice were infected with *H. pylori* strain PM-SS1 for 30 and 60 days to monitor cell infiltration and gastric histopathological changes. On day 30 post-infection, a group of mice were euthanized for baseline immunological measurements and the rest were divided into two groups: one treated with metronidazole and one treated with sterile PBS as a control. These groups were euthanized at day 60 post infection.
(TIF)

Table S1 **Calibration database to adjust parameters on the** ***Helicobacter pylori*** **computational deterministic model.** Data for Th1, Th17, Treg, effector dendritic cells (eDCs), tolerogenic dendritic cells (tDCs), M1 and M2 macrophages is represented as either external inputs for the model or internal readings. The amount of HP (external input) will trigger different phenotype induction depending on the concentration. Consequently, different cell types in the gastric Lamina Propria (LPL) or in the gastric lymph nodes (GLN) will be upregulated or downregulated (internal readings). In the database, ‘TRT’ denotes a control versus an infected mouse, ‘Day’ corresponds to the day where measurements were taken and ‘HP_0′ is the inoculation dose of *H. pylori*. Furthermore, macroscopic scores for the GLN and gastric LP were annotated (Score_GLN and Score_LP) and leukocytic infiltration (LL), mucosal thickening (MT) and epithelial erosion (EE) was measured. Numbers are expressed as absolute cell numbers after exclusion of CD45- cells. A list of phenotypic markers for these populations can be found in Table S3.
(XLSX)

Table S2 **Table of model assumptions.** Table of assumptions were taken biologically or theoretically to ensure proper modulation of species in the immune responses to *Helicobacter pylori* model.
(XLSX)

Table S3 **Immunophenotypic markers used in flow cytometry to characterize immune subsets.** Different immune cell markers were used to characterize Th1, Th17, Treg, M1, M2 and effector and tolerogenic dendritic cells in both gastric lamina propria and gastric lymph nodes.
(XLSX)

Table S4 **Table of model predictions.** Table of prediction computed in the Immune responses to *Helicobacter pylori* model by either the ODE- or ABM-system, as well as how experimental data supports such predictions.
(XLSX)

Table S5 **Evolution of parameter values used in the Agent-Based Model (ABM) and Ordinary Differential Equation (ODE)-based** ***Helicobacter pylori*** **infection models.** Model parameters in the ODE model were obtained from experimental data in mice by running a Particle Swarm algorithm embedded in COPASI. These values were the starting point for optimization methods that would generate the parameter values for the ABM in ENISI. Given that most parameter optimization methods are localized, the starting point is important and can have a significant impact on the chosen estimated parameter value. Thus, narrowing the search using the parameters of the ODE model represents an efficient way to obtain values for ABM models. Furthermore, the semantics of the ABM are slightly different from the ODE model, in part due to inherent assumptions of the ODE model and also how the ENISI software is set up, making the parameter sets different as well.
(XLSX)

Table S6 **Side-by-side comparison table of parameter values used in ENISI.** Parameters were first adjusted using the ODE-based modeling parameters and they were manually adjusted. Parameters for wild-type (WT), T cell specific PPARγ knock-out (T-PPARγ) and myeloid cell-specific PPARγ knock-out (M-PPARγ) are shown.
(XLSX)

## References

[1] Atherton JC, Blaser MJ (2009) Coadaptation of Helicobacter pylori and humans: ancient history, modern implications. J Clin Invest 119: 2475–2487. 19729845 10.1172/JCI38605 PMC2735910

[2] Hitzler I, Kohler E, Engler DB, Yazgan AS, Muller A (2012) The role of Th cell subsets in the control of Helicobacter infections and in T cell-driven gastric immunopathology. Front Immunol 3: 142. 22675328 10.3389/fimmu.2012.00142 PMC3365484

[3] Karttunen R, Karttunen T, Ekre HP, MacDonald TT (1995) Interferon gamma and interleukin 4 secreting cells in the gastric antrum in Helicobacter pylori positive and negative gastritis. Gut 36: 341–345. 7698689 10.1136/gut.36.3.341 PMC1382441

[4] Bamford KB, Fan X, Crowe SE, Leary JF, Gourley WK, et al (1998) Lymphocytes in the human gastric mucosa during Helicobacter pylori have a T helper cell 1 phenotype. Gastroenterology 114: 482–492. 9496938 10.1016/s0016-5085(98)70531-1

[5] Smythies LE, Waites KB, Lindsey JR, Harris PR, Ghiara P, et al (2000) Helicobacter pylori-induced mucosal inflammation is Th1 mediated and exacerbated in IL-4, but not IFN-gamma, gene-deficient mice. J Immunol 165: 1022–1029. 10878379 10.4049/jimmunol.165.2.1022

[6] Afkarian M, Sedy JR, Yang J, Jacobson NG, Cereb N, et al (2002) T-bet is a STAT1-induced regulator of IL-12R expression in naive CD4+ T cells. Nat Immunol 3: 549–557. 12006974 10.1038/ni794

[7] Bettelli E, Carrier Y, Gao W, Korn T, Strom TB, et al (2006) Reciprocal developmental pathways for the generation of pathogenic effector TH17 and regulatory T cells. Nature 441: 235–238. 16648838 10.1038/nature04753

[8] Philipson CW, Bassaganya-Riera J, Viladomiu M, Pedragosa M, Guerrant RL, et al (2013) The role of peroxisome proliferator-activated receptor gamma in immune responses to enteroaggregative Escherichia coli infection. PLoS One 8: e57812. 23469071 10.1371/journal.pone.0057812 PMC3585146

[9] Liang SC, Tan XY, Luxenberg DP, Karim R, Dunussi-Joannopoulos K, et al (2006) Interleukin (IL)-22 and IL-17 are coexpressed by Th17 cells and cooperatively enhance expression of antimicrobial peptides. J Exp Med 203: 2271–2279. 16982811 10.1084/jem.20061308 PMC2118116

[10] Marson A, Kretschmer K, Frampton GM, Jacobsen ES, Polansky JK, et al (2007) Foxp3 occupancy and regulation of key target genes during T-cell stimulation. Nature 445: 931–935. 17237765 10.1038/nature05478 PMC3008159

[11] Ando T, Goto Y, Ishiguro K, Maeda O, Watanabe O, et al (2007) The interaction of host genetic factors and Helicobacter pylori infection. Inflammopharmacology 15: 10–14. 17323188 10.1007/s10787-006-1556-y

[12] Hontecillas R, Bassaganya-Riera J (2007) Peroxisome proliferator-activated receptor gamma is required for regulatory CD4+ T cell-mediated protection against colitis. J Immunol 178: 2940–2949. 17312139 10.4049/jimmunol.178.5.2940

[13] Bassaganya-Riera J, Dominguez-Bello MG, Kronsteiner B, Carbo A, Lu P, et al.. (2012) Helicobacter pylori colonization ameliorates glucose homeostasis in mice through a PPAR gamma-dependent mechanism. PLoS One In Press. 10.1371/journal.pone.0050069 PMC3499487 23166823

[14] Bazargani A, Khoramrooz SS, Kamali-Sarvestani E, Taghavi SA, Saberifiroozi M (2010) Association between peroxisome proliferator-activated receptor-gamma gene polymorphism (Pro12Ala) and Helicobacter pylori infection in gastric carcinogenesis. Scand J Gastroenterol 45: 1162–1167. 20568969 10.3109/00365521.2010.499959

[15] Yao L, Liu F, Sun L, Wu H, Guo C, et al (2010) Upregulation of PPARgamma in tissue with gastric carcinoma. Hybridoma (Larchmt) 29: 341–343. 20715992 10.1089/hyb.2010.0013

[16] Konturek PC, Kania J, Kukharsky V, Raithel M, Ocker M, et al (2004) Implication of peroxisome proliferator-activated receptor gamma and proinflammatory cytokines in gastric carcinogenesis: link to Helicobacter pylori-infection. J Pharmacol Sci 96: 134–143. 15492468 10.1254/jphs.fpj04016x

[17] Son SH, Kim HK, Ji JS, Cho YS, Kim SS, et al (2007) [Expression of peroxisome proliferator-activated receptor (PPAR) gamma in Helicobacter pylori-infected gastric epithelium]. Korean J Gastroenterol 49: 72–78. 17322785

[18] Viladomiu M, Hontecillas R, Pedragosa M, Carbo A, Hoops S, et al (2012) Modeling the role of peroxisome proliferator-activated receptor gamma and microRNA-146 in mucosal immune responses to Clostridium difficile. PLoS One 7: e47525. 23071818 10.1371/journal.pone.0047525 PMC3469550

[19] Carbo A, Hontecillas R, Kronsteiner B, Viladomiu M, Pedragosa M, et al (2013) Systems modeling of molecular mechanisms controlling cytokine-driven CD4+ T cell differentiation and phenotype plasticity. PLoS Comput Biol 9: e1003027. 23592971 10.1371/journal.pcbi.1003027 PMC3617204

[20] Lundgren A, Trollmo C, Edebo A, Svennerholm AM, Lundin BS (2005) Helicobacter pylori-specific CD4+ T cells home to and accumulate in the human Helicobacter pylori-infected gastric mucosa. Infect Immun 73: 5612–5619. 16113278 10.1128/IAI.73.9.5612-5619.2005 PMC1231054

[21] Tan S, Tompkins LS, Amieva MR (2009) Helicobacter pylori usurps cell polarity to turn the cell surface into a replicative niche. PLoS Pathog 5: e1000407. 19412339 10.1371/journal.ppat.1000407 PMC2669173

[22] Naumann M, Crabtree JE (2004) Helicobacter pylori-induced epithelial cell signalling in gastric carcinogenesis. Trends Microbiol 12: 29–36. 14700549 10.1016/j.tim.2003.11.005

[23] Andres S, Schmidt HM, Mitchell H, Rhen M, Maeurer M, et al (2011) Helicobacter pylori defines local immune response through interaction with dendritic cells. FEMS Immunol Med Microbiol 61: 168–178. 21175878 10.1111/j.1574-695X.2010.00761.x

[24] Zhang M, Liu M, Luther J, Kao JY (2010) Helicobacter pylori directs tolerogenic programming of dendritic cells. Gut Microbes 1: 325–329. 21327041 10.4161/gmic.1.5.13052 PMC3023617

[25] Quiding-Jarbrink M, Raghavan S, Sundquist M (2010) Enhanced M1 macrophage polarization in human helicobacter pylori-associated atrophic gastritis and in vaccinated mice. PLoS One 5: e15018. 21124899 10.1371/journal.pone.0015018 PMC2990716

[26] Bimczok D, Grams JM, Stahl RD, Waites KB, Smythies LE, et al (2011) Stromal regulation of human gastric dendritic cells restricts the Th1 response to Helicobacter pylori. Gastroenterology 141: 929–938. 21699795 10.1053/j.gastro.2011.06.006 PMC3163821

[27] Wong BL, Zhu SL, Huang XR, Ma J, Xia HH, et al (2009) Essential role for macrophage migration inhibitory factor in gastritis induced by Helicobacter pylori. Am J Pathol 174: 1319–1328. 19286569 10.2353/ajpath.2009.080708 PMC2671363

[28] Zhuang Y, Shi Y, Liu XF, Zhang JY, Liu T, et al (2011) Helicobacter pylori-infected macrophages induce Th17 cell differentiation. Immunobiology 216: 200–207. 21112468 10.1016/j.imbio.2010.05.005

[29] Hoops S, Sahle S, Gauges R, Lee C, Pahle J, et al (2006) COPASI–a COmplex PAthway SImulator. Bioinformatics 22: 3067–3074. 17032683 10.1093/bioinformatics/btl485

[30] Wendelsdorf K, Alam M, Bassaganya-Riera J, Bisset K, Eubank S, et al (2012) ENteric Immunity SImulator: A tool for in silico study of gastroenteric infections. IEEE Transactions on NanoBioScience 11: 273–288. 22987134 10.1109/TNB.2012.2211891 PMC3715318

[31] Funahashi A, Tanimura N, Morohashi M, Kitano H (2003) CellDesigner: a process diagram editor for gene-regulatory and biochemical networks. BIOSILICO 1: 159–162.

[32] Iwasaki A (2007) Mucosal dendritic cells. Annu Rev Immunol 25: 381–418. 17378762 10.1146/annurev.immunol.25.022106.141634

[33] Ng SC, Kamm MA, Stagg AJ, Knight SC (2010) Intestinal dendritic cells: their role in bacterial recognition, lymphocyte homing, and intestinal inflammation. Inflamm Bowel Dis 16: 1787–1807. 20222140 10.1002/ibd.21247

[34] Littman DR, Rudensky AY (2010) Th17 and regulatory T cells in mediating and restraining inflammation. Cell 140: 845–858. 20303875 10.1016/j.cell.2010.02.021

[35] Kennedy J (1997) The particle swarm: social adaptation of knowledge. IEEE Congress on Evolutionary Computation: 303–308.

[36] Hoops S, Sahle S, Gauges R, Lee C, Pahle J, et al (2006) COPASI–a complex pathway simulator. Bioinformatics 22: 3067–3074. 17032683 10.1093/bioinformatics/btl485

[37] Spiegelman BM (1998) PPARgamma in monocytes: less pain, any gain? Cell 93: 153–155. 9568708 10.1016/s0092-8674(00)81567-6

[38] Klotz L, Burgdorf S, Dani I, Saijo K, Flossdorf J, et al (2009) The nuclear receptor PPAR gamma selectively inhibits Th17 differentiation in a T cell-intrinsic fashion and suppresses CNS autoimmunity. J Exp Med 206: 2079–2089. 19737866 10.1084/jem.20082771 PMC2757877

[39] Mansen A, Guardiola-Diaz H, Rafter J, Branting C, Gustafsson JA (1996) Expression of the peroxisome proliferator-activated receptor (PPAR) in the mouse colonic mucosa. Biochem Biophys Res Commun 222: 844–851. 8651933 10.1006/bbrc.1996.0832

[40] Wohlfert EA, Nichols FC, Nevius E, Clark RB (2007) Peroxisome proliferator-activated receptor gamma (PPARgamma) and immunoregulation: enhancement of regulatory T cells through PPARgamma-dependent and -independent mechanisms. J Immunol 178: 4129–4135. 17371968 10.4049/jimmunol.178.7.4129

[41] Adria Carbo RH, Kronsteiner B, Viladomiu M, Pedragosa M, Lu P, et al.. (2013) Systems modeling of molecular mechanisms controlling cytokine-driven CD4+ T cell differentiation and phenotype plasticity. PLoS Comput Biol In Press. 10.1371/journal.pcbi.1003027 PMC3617204 23592971

[42] Bisset KR, Alam MM, Bassaganya-Riera J, Carbo A, Eubank S, et al. High-Performance Interaction-Based Simulation of Gut Immunopathologies with ENteric Immunity Simulator (ENISI); 2012. IEEE Computer Society. 48–59.

[43] Olokoba AB, Obateru OA, Bojuwoye MO (2013) Helicobacter pylori eradication therapy: A review of current trends. Niger Med J 54: 1–4. 23661891 10.4103/0300-1652.108884 PMC3644737

[44] Parsonnet J, Hansen S, Rodriguez L, Gelb AB, Warnke RA, et al (1994) Helicobacter pylori infection and gastric lymphoma. N Engl J Med 330: 1267–1271. 8145781 10.1056/NEJM199405053301803

[45] Parsonnet J, Isaacson PG (2004) Bacterial infection and MALT lymphoma. N Engl J Med 350: 213–215. 14724298 10.1056/NEJMp038200

[46] Correa P, Houghton J (2007) Carcinogenesis of Helicobacter pylori. Gastroenterology 133: 659–672. 17681184 10.1053/j.gastro.2007.06.026

[47] Blaser MJ (2008) Disappearing microbiota: Helicobacter pylori protection against esophageal adenocarcinoma. Cancer Prev Res (Phila Pa) 1: 308–311. 10.1158/1940-6207.CAPR-08-0170 19138974

[48] Vieth M, Masoud B, Meining A, Stolte M (2000) Helicobacter pylori infection: protection against Barrett’s mucosa and neoplasia? Digestion 62: 225–231. 11070405 10.1159/000007820

[49] Vaezi MF, Falk GW, Peek RM, Vicari JJ, Goldblum JR, et al (2000) CagA-positive strains of Helicobacter pylori may protect against Barrett’s esophagus. Am J Gastroenterol 95: 2206–2211. 11007219 10.1111/j.1572-0241.2000.02305.x

[50] Chow WH, Blaser MJ, Blot WJ, Gammon MD, Vaughan TL, et al (1998) An inverse relation between cagA+ strains of Helicobacter pylori infection and risk of esophageal and gastric cardia adenocarcinoma. Cancer Res 58: 588–590. 9485003

[51] Blaser MJ, Chen Y, Reibman J (2008) Does Helicobacter pylori protect against asthma and allergy? Gut. 10.1136/gut.2007.133462 PMC3888205 18194986

[52] Chen Y, Blaser MJ (2007) Inverse associations of Helicobacter pylori with asthma and allergy. Arch Intern Med 167: 821–827. 17452546 10.1001/archinte.167.8.821

[53] Lang L (2007) Childhood acquisition of Helicobacter pylori linked to reduced asthma and allergy risk. Gastroenterology 133: 6. 17631119 10.1053/j.gastro.2007.05.011

[54] McCune A, Lane A, Murray L, Harvey I, Nair P, et al (2003) Reduced risk of atopic disorders in adults with Helicobacter pylori infection. Eur J Gastroenterol Hepatol 15: 637–640. 12840675 10.1097/00042737-200306000-00010

[55] Harris PR, Wright SW, Serrano C, Riera F, Duarte I, et al (2008) Helicobacter pylori gastritis in children is associated with a regulatory T-cell response. Gastroenterology 134: 491–499. 18242215 10.1053/j.gastro.2007.11.006

[56] Jang TJ (2010) The number of Foxp3-positive regulatory T cells is increased in Helicobacter pylori gastritis and gastric cancer. Pathol Res Pract 206: 34–38. 19819643 10.1016/j.prp.2009.07.019

[57] Esplugues E, Huber S, Gagliani N, Hauser AE, Town T, et al (2011) Control of TH17 cells occurs in the small intestine. Nature 475: 514–518. 21765430 10.1038/nature10228 PMC3148838

[58] Sayi A, Kohler E, Hitzler I, Arnold I, Schwendener R, et al (2009) The CD4+ T cell-mediated IFN-gamma response to Helicobacter infection is essential for clearance and determines gastric cancer risk. J Immunol 182: 7085–7101. 19454706 10.4049/jimmunol.0803293

[59] Shi Y, Liu XF, Zhuang Y, Zhang JY, Liu T, et al (2010) Helicobacter pylori-induced Th17 responses modulate Th1 cell responses, benefit bacterial growth, and contribute to pathology in mice. J Immunol 184: 5121–5129. 20351183 10.4049/jimmunol.0901115

[60] Algood HM, Allen SS, Washington MK, Peek RM Jr, Miller GG, et al (2009) Regulation of gastric B cell recruitment is dependent on IL-17 receptor A signaling in a model of chronic bacterial infection. J Immunol 183: 5837–5846. 19812196 10.4049/jimmunol.0901206 PMC2834183

[61] O’Connor W Jr, Zenewicz LA, Flavell RA (2010) The dual nature of T(H)17 cells: shifting the focus to function. Nat Immunol 11: 471–476. 20485275 10.1038/ni.1882

[62] Quiding-Jarbrink M, Lundin BS, Lonroth H, Svennerholm AM (2001) CD4+ and CD8+ T cell responses in Helicobacter pylori-infected individuals. Clin Exp Immunol 123: 81–87. 11168002 10.1046/j.1365-2249.2001.01427.x PMC1905955

[63] Krakowka S, Ringler SS, Eaton KA, Green WB, Leunk R (1996) Manifestations of the local gastric immune response in gnotobiotic piglets infected with Helicobacter pylori. Vet Immunol Immunopathol 52: 159–173. 8809998 10.1016/0165-2427(95)05547-9

[64] Ricote M, Li AC, Willson TM, Kelly CJ, Glass CK (1998) The peroxisome proliferator-activated receptor-gamma is a negative regulator of macrophage activation. Nature 391: 79–82. 9422508 10.1038/34178

[65] Wu L, Yan C, Czader M, Foreman O, Blum JS, et al (2012) Inhibition of PPARgamma in myeloid-lineage cells induces systemic inflammation, immunosuppression, and tumorigenesis. Blood 119: 115–126. 22053106 10.1182/blood-2011-06-363093 PMC3251224

[66] Abdullah Z, Geiger S, Nino-Castro A, Bottcher JP, Muraliv E, et al (2012) Lack of PPARgamma in myeloid cells confers resistance to Listeria monocytogenes infection. PLoS One 7: e37349. 22629382 10.1371/journal.pone.0037349 PMC3357414

[67] Zhang MA, Rego D, Moshkova M, Kebir H, Chruscinski A, et al (2012) Peroxisome proliferator-activated receptor (PPAR)alpha and -gamma regulate IFNgamma and IL-17A production by human T cells in a sex-specific way. Proc Natl Acad Sci U S A 109: 9505–9510. 22647601 10.1073/pnas.1118458109 PMC3386070

[68] Bassaganya-Riera J, Viladomiu M, Pedragosa M, De Simone C, Hontecillas R (2012) Immunoregulatory mechanisms underlying prevention of colitis-associated colorectal cancer by probiotic bacteria. PLoS One 7: e34676. 22511958 10.1371/journal.pone.0034676 PMC3325233

[69] Klotz L, Knolle P (2011) Nuclear receptors: TH17 cell control from within. FEBS Lett 585: 3764–3769. 21745474 10.1016/j.febslet.2011.06.027

[70] Lei J, Hasegawa H, Matsumoto T, Yasukawa M (2010) Peroxisome proliferator-activated receptor alpha and gamma agonists together with TGF-beta convert human CD4+CD25− T cells into functional Foxp3+ regulatory T cells. J Immunol 185: 7186–7198. 21057085 10.4049/jimmunol.1001437

[71] Hoechst B, Gamrekelashvili J, Manns MP, Greten TF, Korangy F (2011) Plasticity of human Th17 cells and iTregs is orchestrated by different subsets of myeloid cells. Blood 117: 6532–6541. 21493801 10.1182/blood-2010-11-317321

[72] Kryczek I, Wu K, Zhao E, Wei S, Vatan L, et al (2011) IL-17+ regulatory T cells in the microenvironments of chronic inflammation and cancer. J Immunol 186: 4388–4395. 21357259 10.4049/jimmunol.1003251

[73] Nagai S, Mimuro H, Yamada T, Baba Y, Moro K, et al (2007) Role of Peyer’s patches in the induction of Helicobacter pylori-induced gastritis. Proc Natl Acad Sci U S A 104: 8971–8976. 17502608 10.1073/pnas.0609014104 PMC1885612

