# Supplementary figures and images for: Predictive Computational Modeling of the Mucosal Immune Responses during Helicobacter pylori Infection
Source: PLoS One. 2013 Sep 5;8(9):e73365. doi: 10.1371/journal.pone.0073365 (PMC3764126; doi:10.1371/journal.pone.0073365)

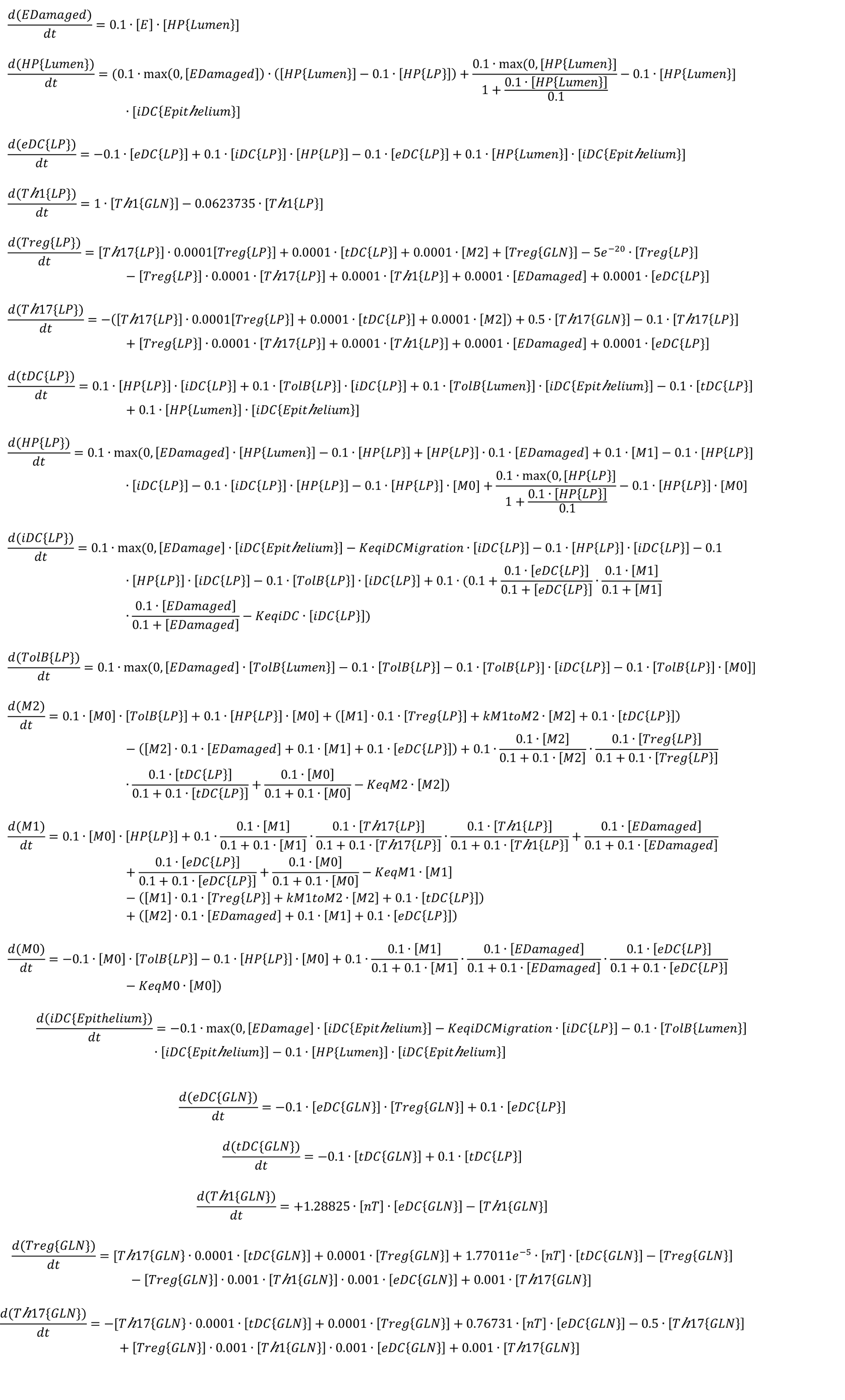

Supplement: Figure S1 — Ordinary Differential Equations (ODE) triggering activation and inhibition regulatory and effector pathways in our H. pylori infection model. Briefly, mass action and contact dependent functions were used to reproduce H. pylori infection cell behaviors in silico based on the addition of H. pylori in the gastric lumen. (TIF) [file pone.0073365.s001.tif]

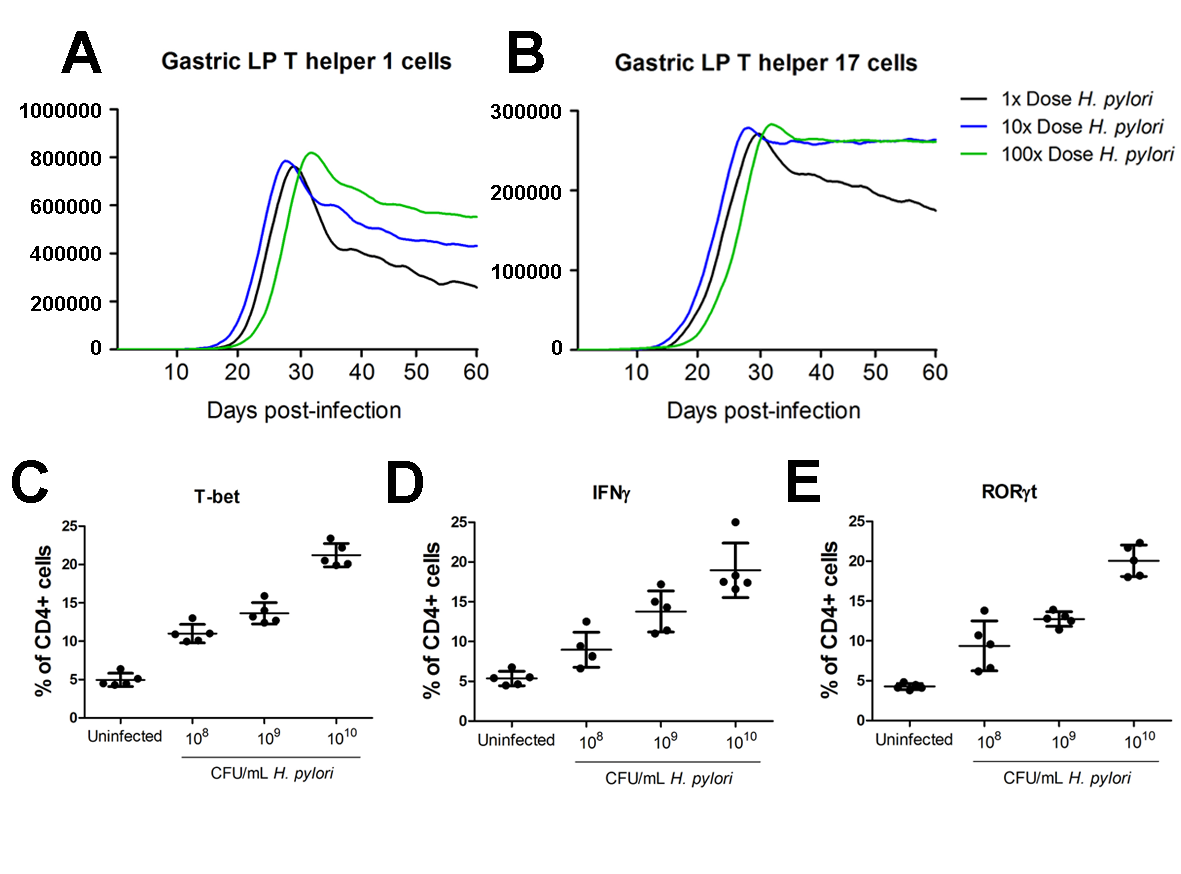

Supplement: Figure S2 — T helper (Th)1 and Th17 responses during Helicobacter pylori infection are dose-dependent. Computational simulations with the H. pylori model demonstrating a dose-response effect with the initial dose of H. pylori in (A) Th1 and (B) Th17. In vivo experimentation validating this prediction by observing increased levels of splenic (A) T-bet, (B) IFNγ and (C) RORγt with increasing concentrations in the initial dose of H. pylori. (TIF) [file pone.0073365.s002.tif]

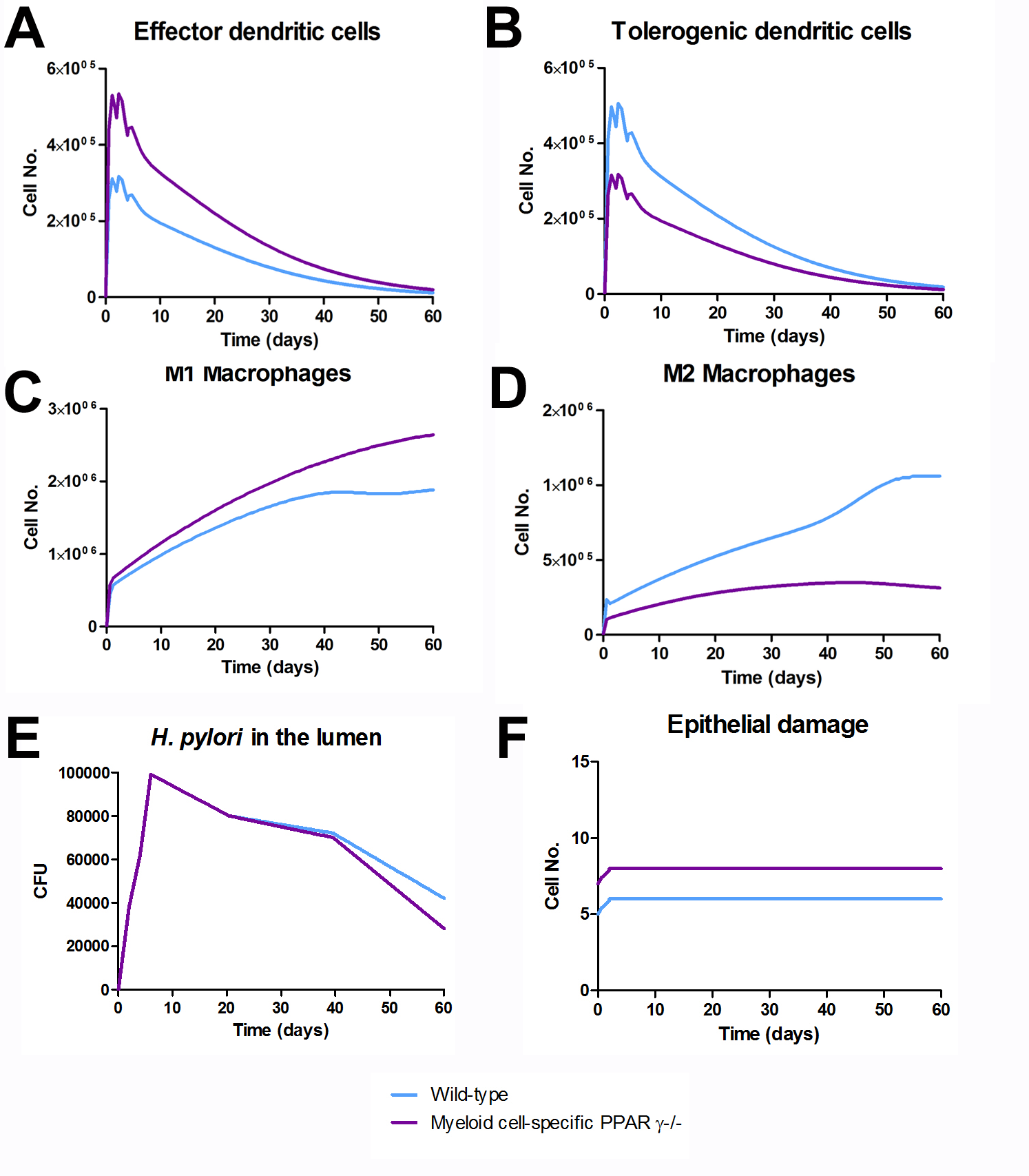

Supplement: Figure S3 — Predicted dynamics of gastric mucosal cell subsets and luminal Helicobacter pylori counts following in silico infection of wild-type and myeloid-specific peroxisome proliferator-activated receptor γ (PPARγ) knockout models. Time-courses were run in silico with infections using 5×10^7^ colony forming units (CFU) of H. pylori to determine myeloid subsets dynamics. The blue lines represent the wild-type model whereas violet lines represent the PPARγ knockout model in (A) gastric lamina propria (LP) effector dendritic cells, (B) LP tolerogenic dendritic cells, (C) LP M1 macrophages, (D) LP M2 macrophages, (E) H. pylori loads in the stomach lumen and (F) epithelial cell damage following infection with H. pylori. (TIF) [file pone.0073365.s003.tif]

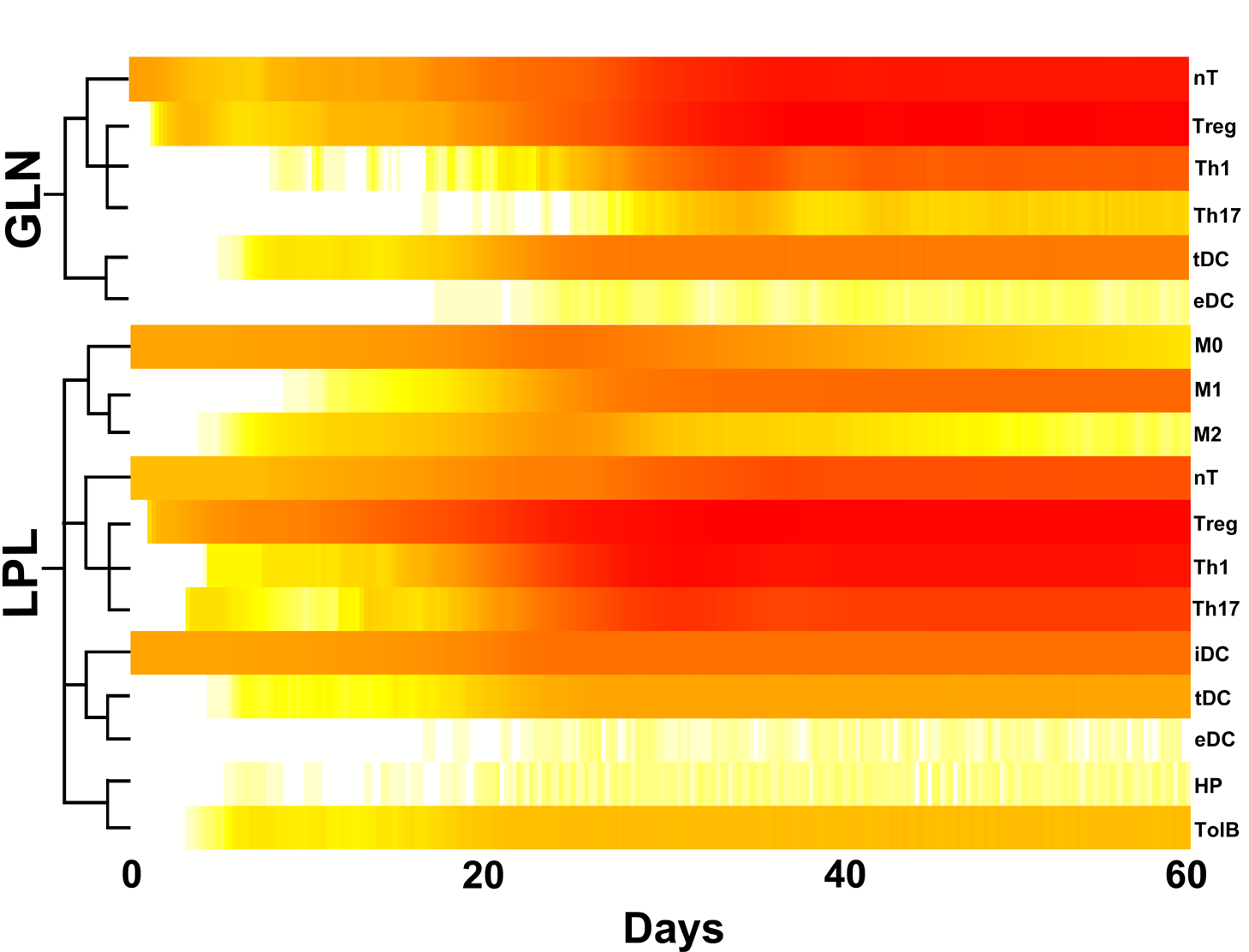

Supplement: Figure S4 — Enteric Immunity Simulator (ENISI) output results after Helicobacter pylori infection in silico . The H.pylori-implemented agent-based model was run as a time-course for 60 days. Heatmap representation of cell concentrations being modulated over time in the gastric Lamina Propria (LPL) and in the Gastric Lymph Nodes (GLN). Cell types are grouped by function and effector vs. regulatory mechanisms. (TIF) [file pone.0073365.s004.tif]

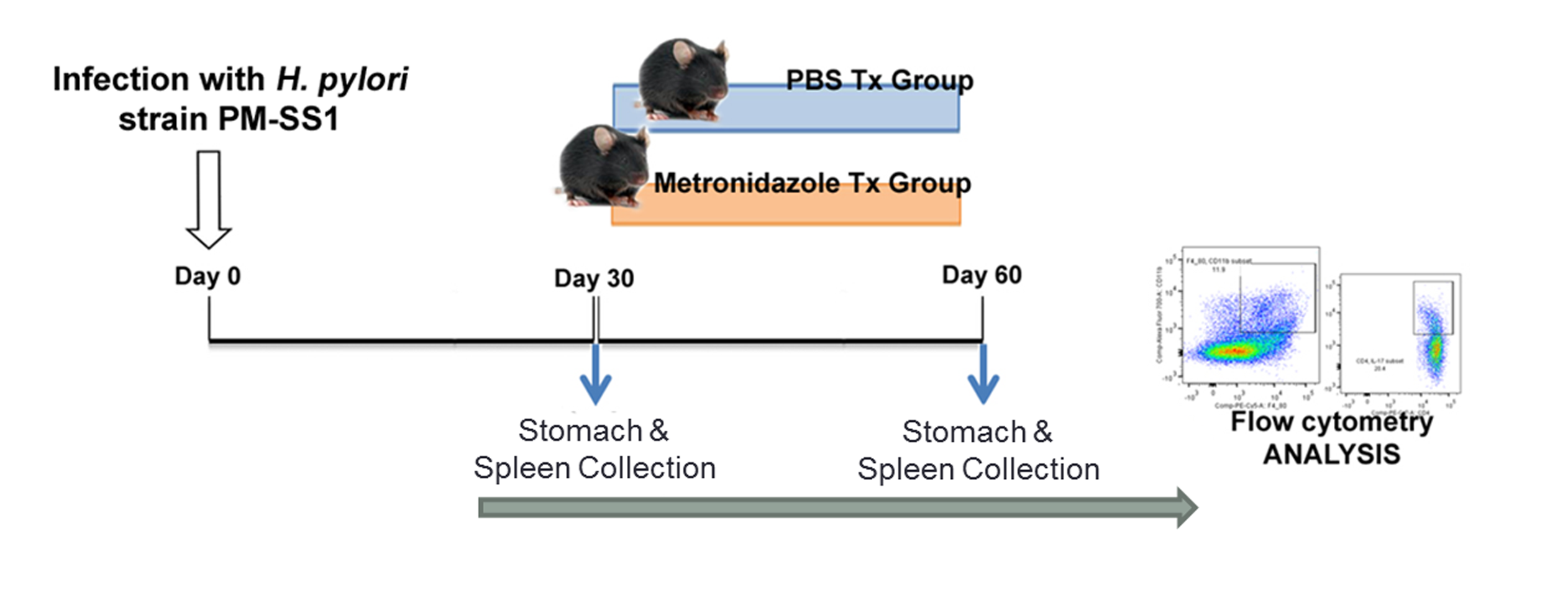

Supplement: Figure S5 — Experimental design to validate model prediction on main inducers of histopathologcal changes during Helicobacter pylori infection. Wild-type mice were infected with H. pylori strain PM-SS1 for 30 and 60 days to monitor cell infiltration and gastric histopathological changes. On day 30 post-infection, a group of mice were euthanized for baseline immunological measurements and the rest were divided into two groups: one treated with metronidazole and one treated with sterile PBS as a control. These groups were euthanized at day 60 post infection. (TIF) [file pone.0073365.s005.tif]
